# Supplementary material for: Reducing the metabolic burden of rRNA synthesis promotes healthy longevity in Caenorhabditis elegans
Source: Nat Commun. 2024 Feb 24;15:1702. doi: 10.1038/s41467-024-46037-w (PMC10894287; doi:10.1038/s41467-024-46037-w)
Supplement: Supplementary file 1 — Supplementary Information [file 41467_2024_46037_MOESM1_ESM.pdf]

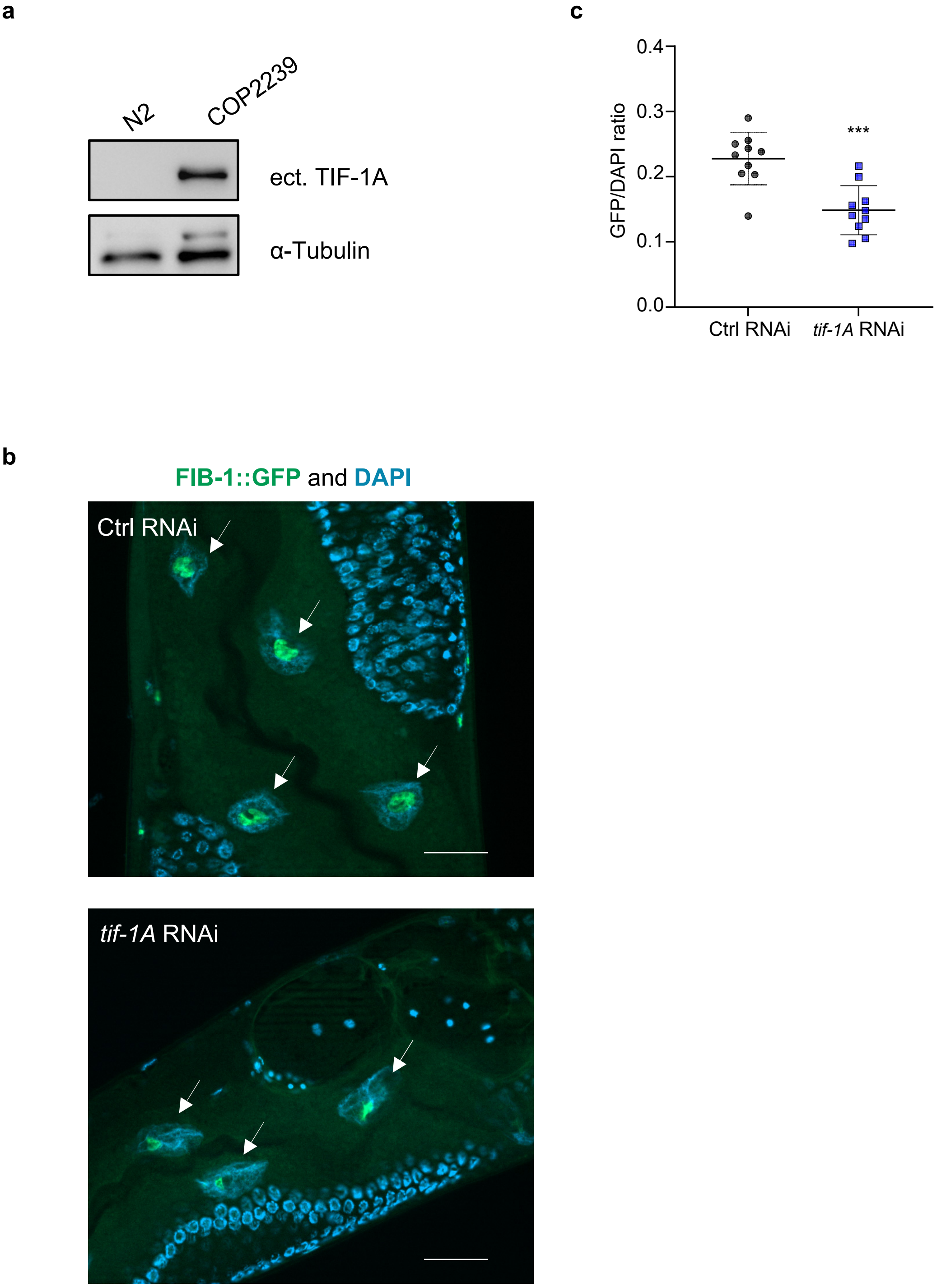

Supplementary Fig. 1 | Cellular effects of the curtailed pre-rRNA synthesis. **a**, Western blot showing expression of ectopic TIF-1A (ect. TIF-1A) in the *C. elegans* strain COP2239. Total protein samples from wild-type (N2 Bristol strain) and COP2239 worms were probed with antibodies against either the Ty1 epitope fused to ect. TIF-1A (upper panel), or α-Tubulin (lower panel). Representative result of n = 3 independent trials is shown. **b**, **c**, Fluorescent microscopy of *C. elegans* strain COP262 expressing the nucleolar marker protein FIB-1::GFP treated with either control (Ctrl) RNAi or *tif-1A* RNAi. Nucleolar size relative to nuclear size was assessed by FIB-1::GFP fluorescence and DNA staining with 4′,6-diamidino-2-phenylindole (DAPI), respectively. Representative images **(b)** and quantification of the nucleolus/nucleus size ratio **(c)**, n = 10 worms per condition were analyzed. Arrows indicate cells with clear FIB-1::GFP fluorescence that were used for the analysis. Scale bar in (**b**) is 20μm. Mean ± S.D. values are shown. P value in **c** was determined with a Student's unpaired t-test and is two-sided. ***P < 0.001. The exact P value and statistical analysis are reported in the Source Data file.

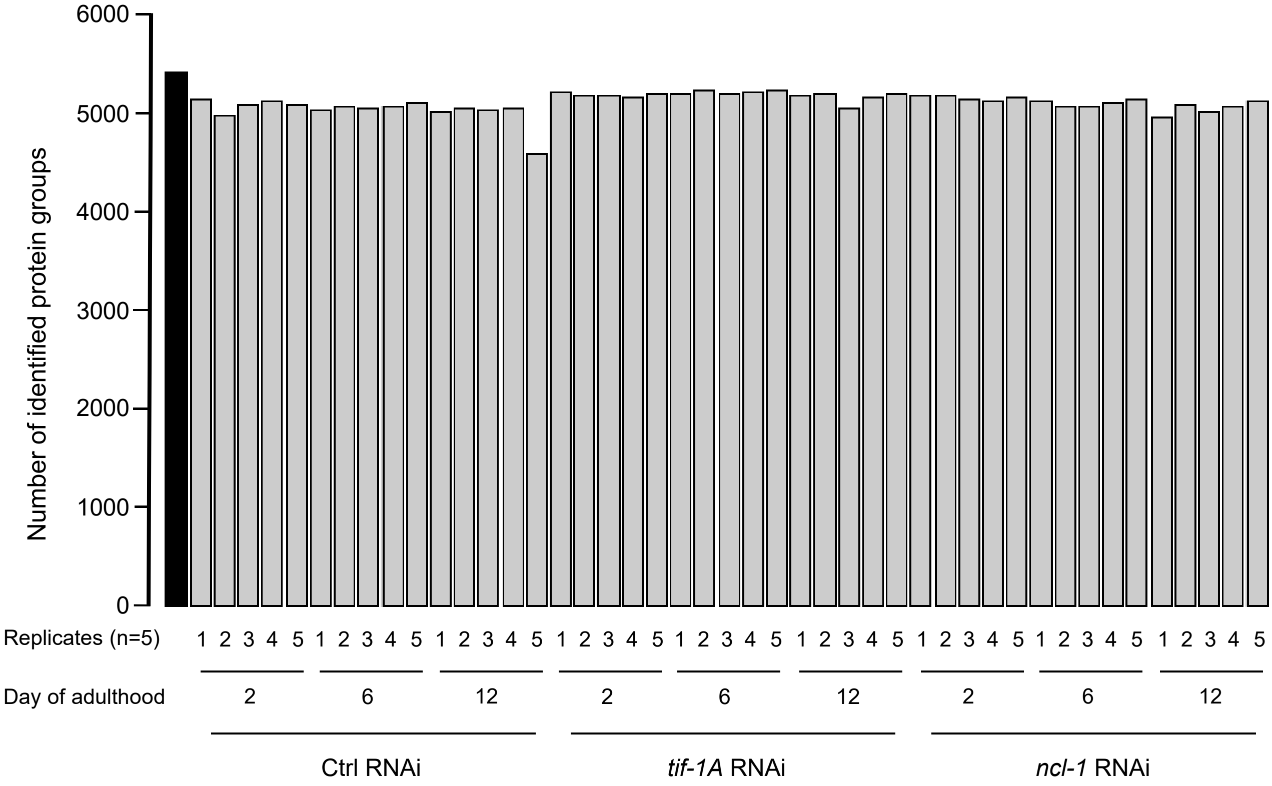

Supplementary Fig. 2 | The depth of proteomics data acquisition. Proteomic analysis of nematodes exposed to Ctrl RNAi, *tif-1A* RNAi or *ncl-1* RNAi was performed using 5 biological replicates per condition. Each replicate consisted of n = 700 worms. The number of protein groups identified in each replicate is shown. The black bar on the left indicates total number of proteins measured in the whole experiment. The corresponding raw data can be found in Supplementary Data 1.

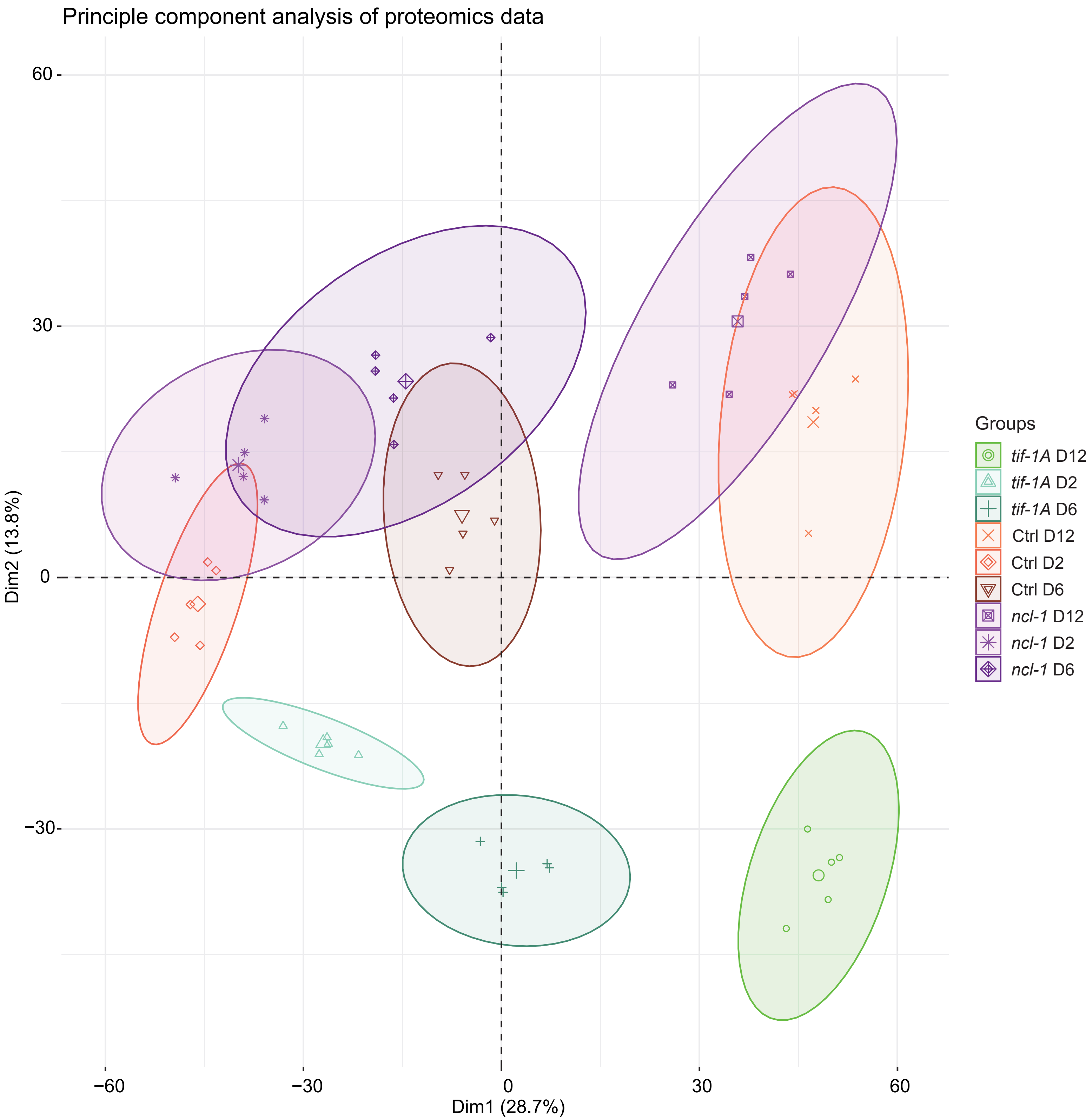

Supplementary Fig. 3 | Influence of Pol I transcriptional activity on aging-dependent proteome dynamics. Proteomes of nematodes treated with Ctrl RNAi, *tif-1A* RNAi or *ncl-1* RNAi were analyzed by mass spectrometry on AD2, AD6 and AD12 in five biological replicates. n = 700 worms per replicate were measured. The 2D whole proteome principle component analysis (PCA) of all tested conditions is shown. The corresponding raw data can be found in Supplementary Data 1.

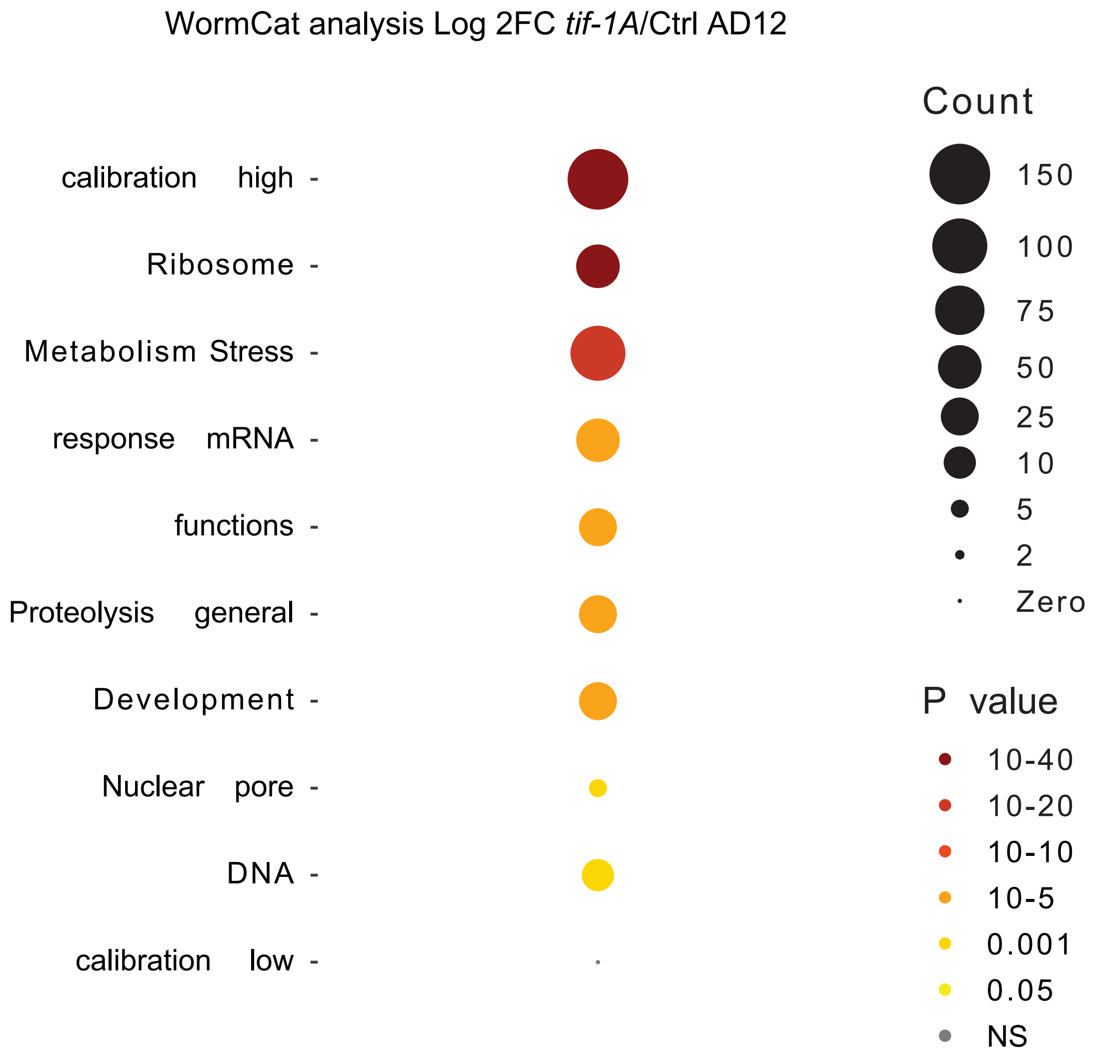

Supplementary Fig. 4 | Ribosome and metabolic functions are enriched among proteome changes elicited by *tif-1A* RNAi treatment during aging. Proteins differentially regulated (log2 fold change>0.58, Q<0.05) between *tif-1A* RNAi and ctrl RNAi treated animals at old age (AD12) were assessed by WormCat to highlight the biological processes modulated by *tif-1A* knockdown during aging. The raw data and analysis are shown in Supplementary Data 4 and 5.

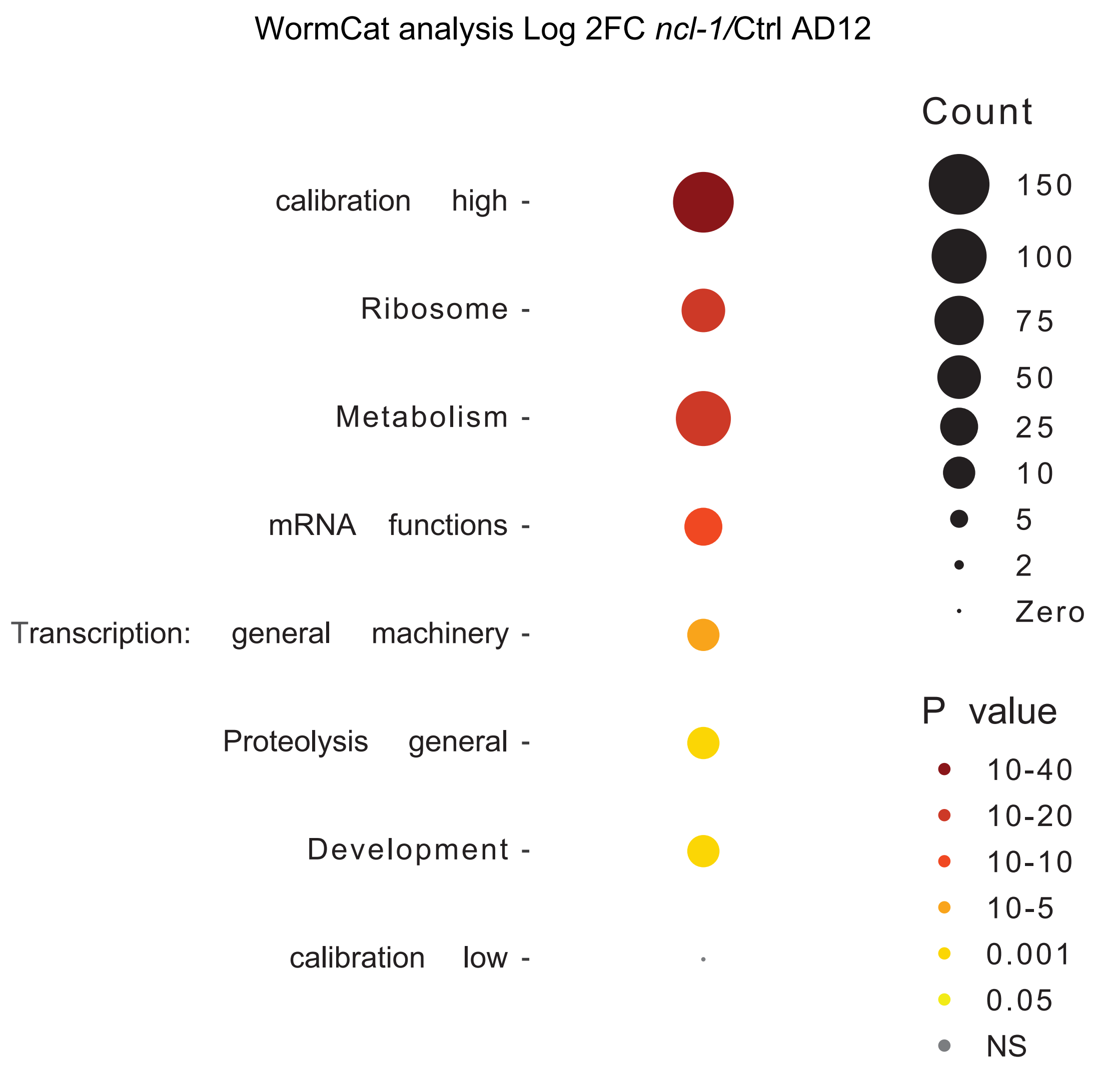

Supplementary Fig. 5 | | Ribosome and metabolic functions are enriched among proteome changes induced by *ncl-1* gene knockdown during aging. Proteins differentially regulated (log2 fold change>0.58, Q<0.05) between *ncl-1* RNAi and ctrl RNAi treated animals at old age (AD12) were assessed by WormCat to highlight the biological processes modulated by *ncl-1* gene knockdown during aging. The raw data and analysis are shown in Supplementary Data 6 and 7.

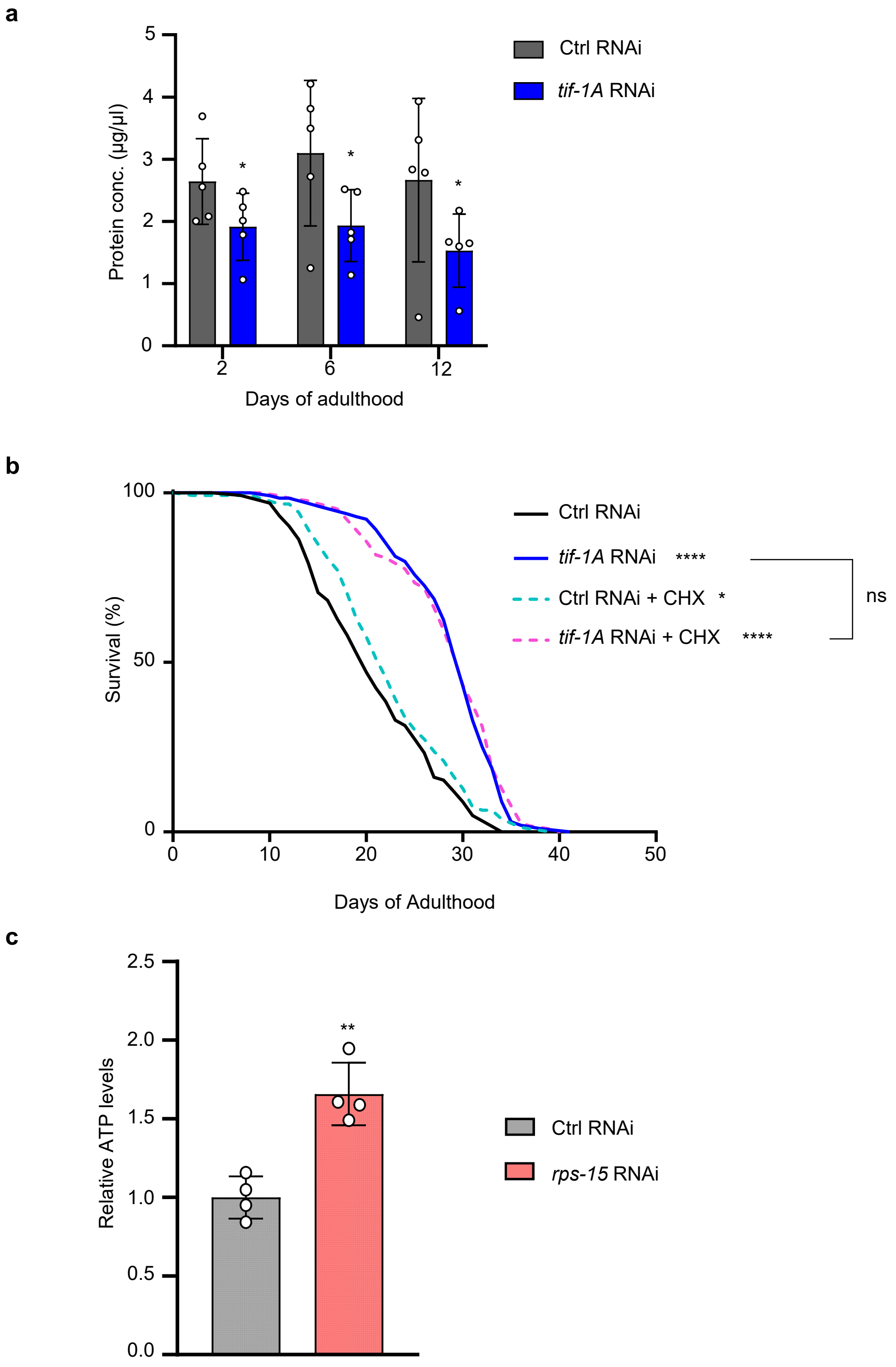

Supplementary Fig. 6 | Longevity effects of the disrupted ribosome biogenesis and pre-rRNA synthesis include translation inhibition and energy saving. a, Measurement of total protein concentration per lysate of worms treated with Ctrl RNAi or *tif-1A* RNAi and harvested at the indicated time points. Each condition has five biological replicates of n = 700 worms. Mean ± S.D. values are displayed. Statistics was computed by an unpaired t-test. **b**, Survival analysis of wild-type animals treated with Ctrl RNAi or *tif-1A* RNAi in the absence or presence of 1 µM cycloheximide (CHX). n = 140 worms per treatment. P values were determined using a Mantel-Cox test. Representative result of 3 independent trials. **c,** ATP measurement in nematodes exposed to Ctrl RNAi or *rps-15* RNAi at 20 °C. Data from 4 independent replicates with n = 50 animals per treatment. Mean ± S.D. values are shown. Student's unpaired t-test was used for statistical analysis. ****P < 0.0001, ** P <0.01, *P < 0.05. All statistical tests used were two-sided, the exact P values and statistical analyses are reported in the Source Data file.

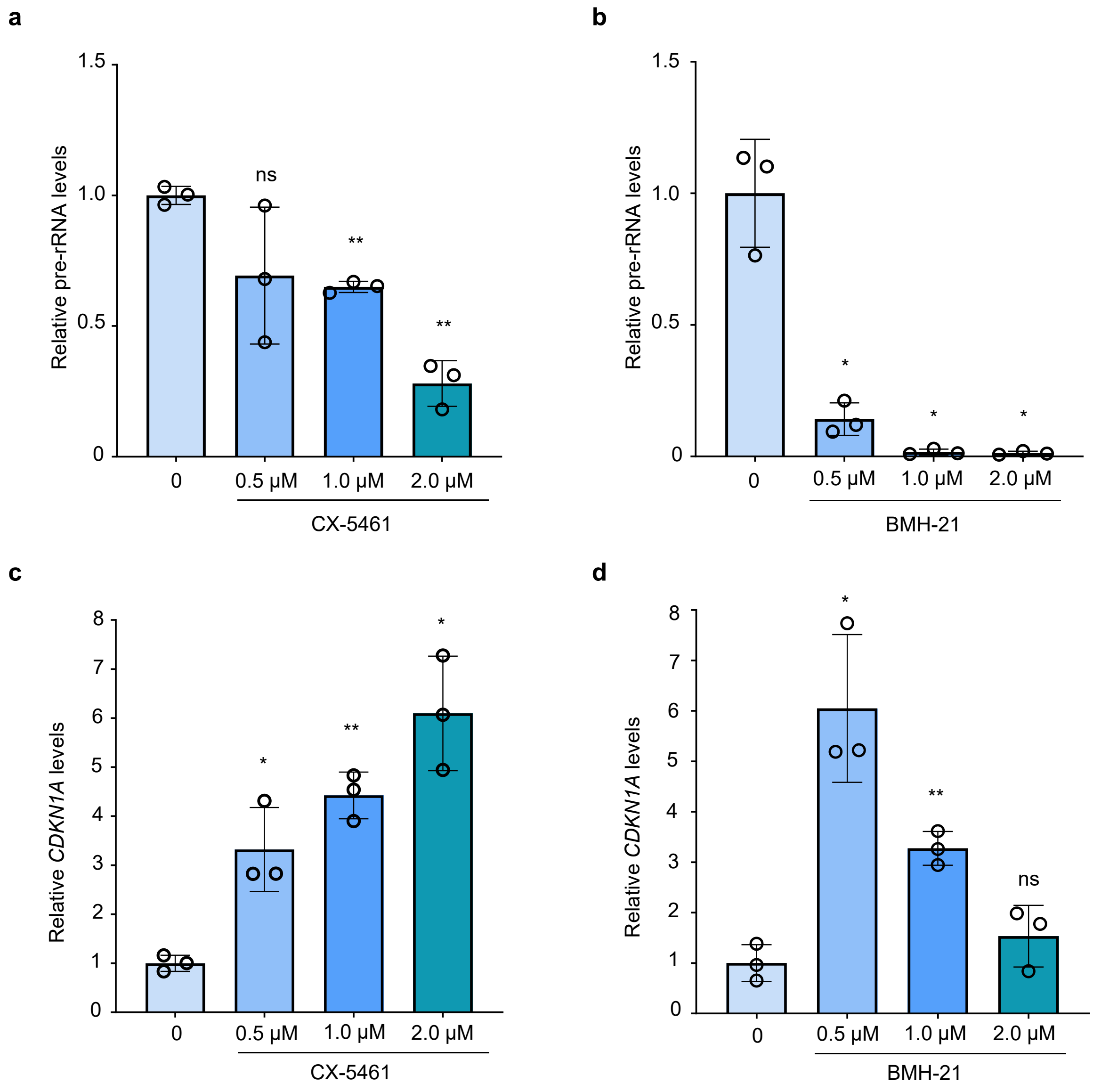

Supplementary Fig. 7 | Pharmacological Pol I inhibitors cause p21 induction in human fibroblasts. Human primary skin fibroblasts (BJ cells) treated with 0.5 µM, 1 µM or 2 µM CX-5461 (**a**, **c**) or 0.5 µM, 1 µM or 2 µM BMH-21 (**b, d**) and assessed for pre-rRNA levels (**a**, **b**) or *CDKN1A* mRNA (encoding p21) levels (**c**, **d**) normalized to *Rpl3* mRNA. Data are displayed relative to the untreated control (set as 1) and are shown as mean ± S.D. of three independent experiments. *P* values were computed with Student's paired *t*-test. ns= not significant, ** *P* <0.01, * *P* <0.05. All statistical tests used were two-sided, the exact *P* values and statistical analyses are reported in the Source Data file.

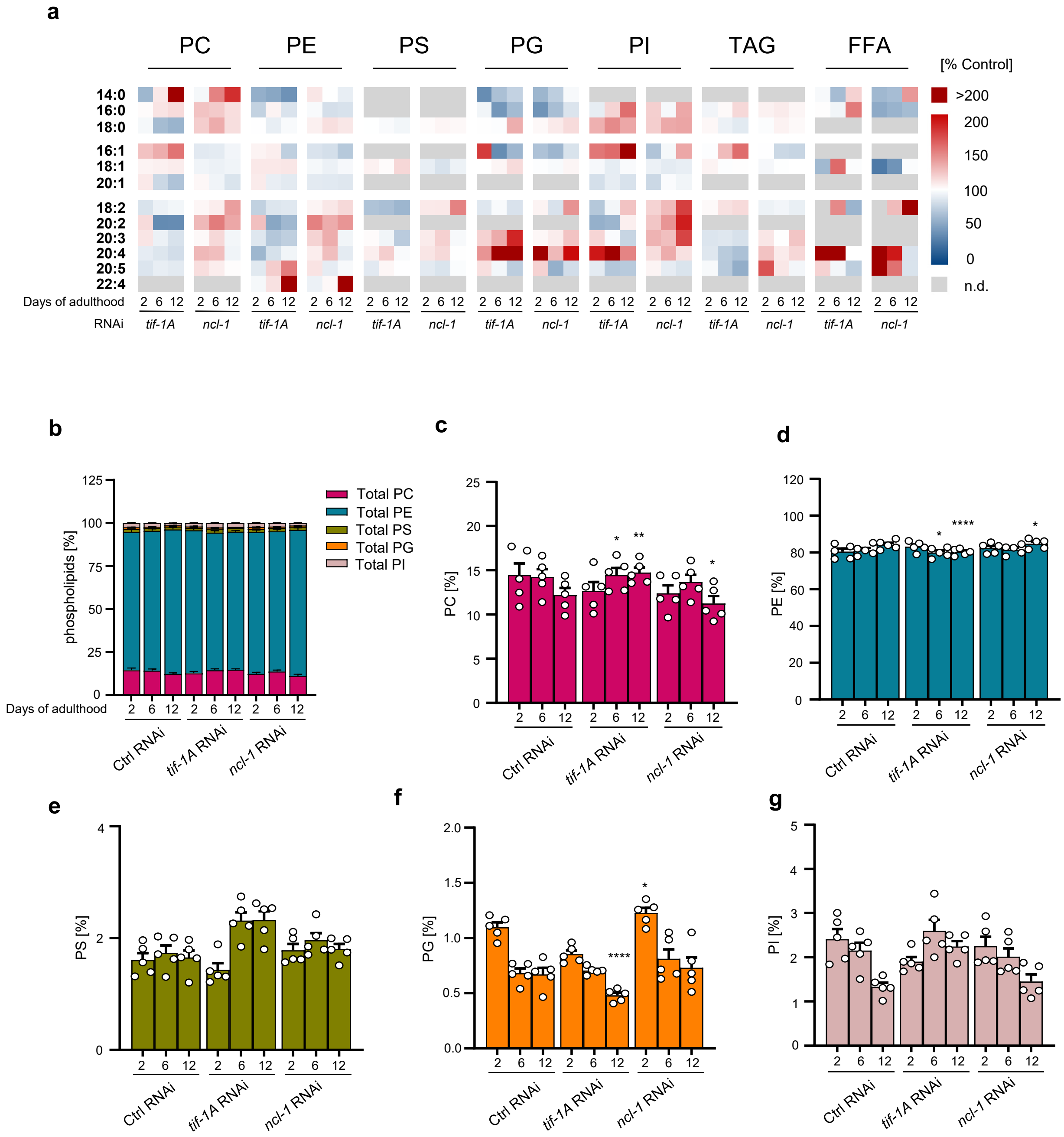

Supplementary Fig. 8 | Modulation of Pol I activity elicits lipidome changes associated with longevity and aging. a, Heatmap showing relative abundances of distinct fatty acids across lipid classes of animals treated with Ctrl, *tif-1A* or *ncl-1* RNAi. Mean values were calculated from relative intensities and presented as % relative to the control of the respective age. **b**, Stacked bar chart of detected phospholipids (mean ± SEM in each case), calculated as % of total phospholipid amount for each condition (using absolute values normalized to the internal standard 1,2-dimyristoyl-sn-glycero-3-phosphatidylcholine, DMPC). **c-g**, Single bar charts of phospholipid classes shown in b, mean ± SEM: **c**, phosphatidylcholine (PC). **d**, phosphatidylethanolamine (PE). **e**, phosphatidylserine (PS). **f**, phosphatidylglycerol (PG). **g**, phosphatidylinositol (PI). Statistical analysis was carried out by two-way ANOVA with Dunnett's *post hoc* test. Only significant *P* values are displayed. In **a-g** each condition has five biological replicates of n = 700 worms. ****P < 0.0001, ** P <0.01, * P <0.05. The raw data and analyses associated with this figure are displayed in Supplementary Data 16 and 17. All statistical tests used were two-sided, the exact *P* values and statistical analyses are reported in the Source Data file.

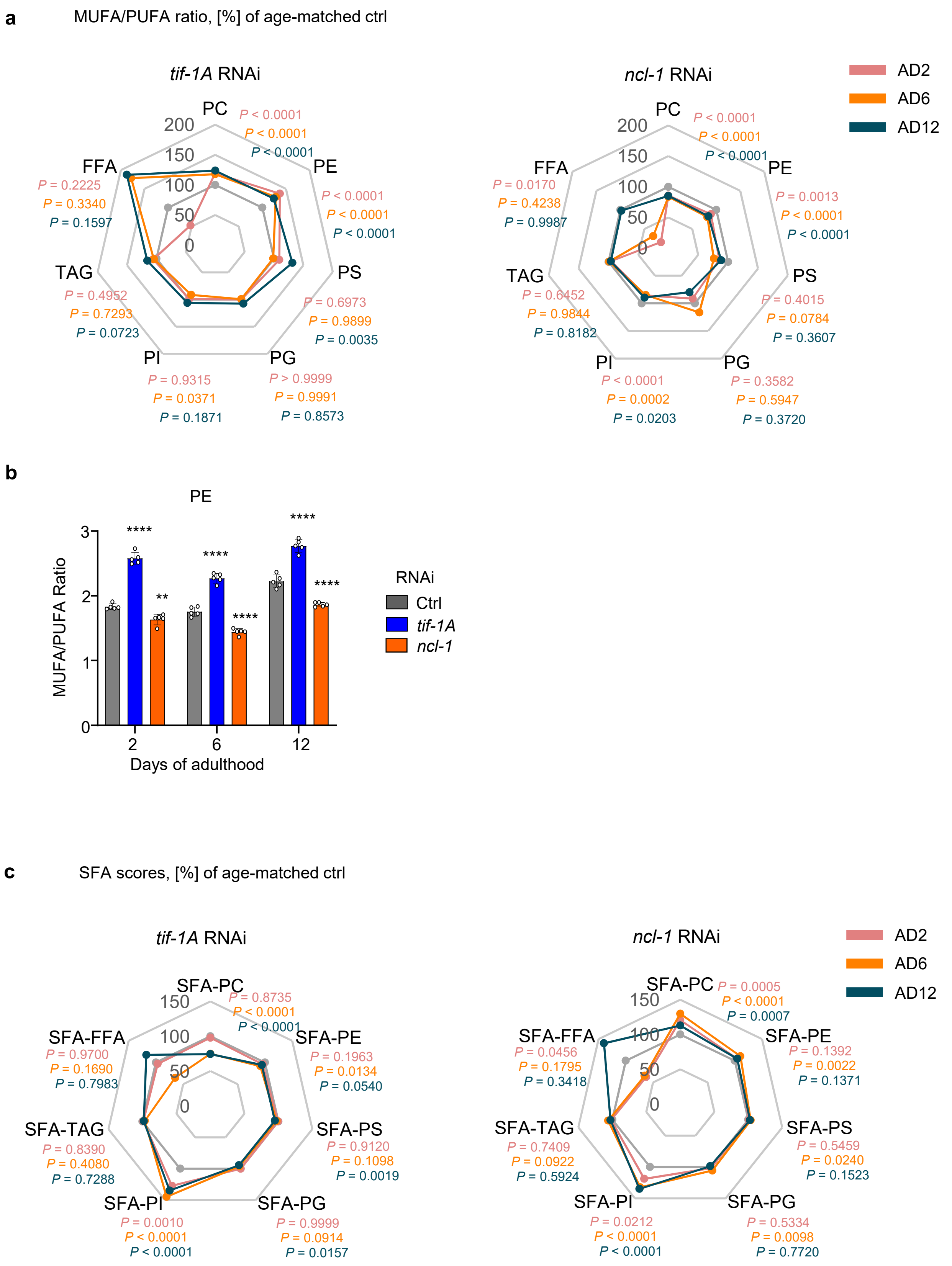
a
MUFA/PUFA ratio, [%] of age-matched ctrl
tif-1A RNAi
ncl-1 RNAi
AD2
AD6
AD12
PC
PE
PS
PG
PI
TAG
FFA
200
150
100
50
0
tif-1A RNAi: PC P < 0.0001, P < 0.0001, P < 0.0001
PE P < 0.0001, P < 0.0001, P < 0.0001
PS P = 0.6973, P = 0.9899, P = 0.0035
PG P > 0.9999, P = 0.9991, P = 0.8573
PI P = 0.9315, P = 0.0371, P = 0.1871
TAG P = 0.4952, P = 0.7293, P = 0.0723
FFA P = 0.2225, P = 0.3340, P = 0.1597
ncl-1 RNAi: PC P < 0.0001, P < 0.0001, P < 0.0001
PE P = 0.0013, P < 0.0001, P < 0.0001
PS P = 0.4015, P = 0.0784, P = 0.3607
PG P = 0.3582, P = 0.5947, P = 0.3720
PI P < 0.0001, P = 0.0002, P = 0.0203
TAG P = 0.6452, P = 0.9844, P = 0.8182
FFA P = 0.0170, P = 0.4238, P = 0.9987
b
PE
MUFA/PUFA Ratio
Days of adulthood
2
6
12
RNAi
Ctrl
tif-1A
ncl-1
****
**
c
SFA scores, [%] of age-matched ctrl
tif-1A RNAi
ncl-1 RNAi
SFA-PC
SFA-PE
SFA-PS
SFA-PG
SFA-PI
SFA-TAG
SFA-FFA
tif-1A RNAi: SFA-PC P = 0.8735, P < 0.0001, P < 0.0001
SFA-PE P = 0.1963, P = 0.0134, P = 0.0540
SFA-PS P = 0.9120, P = 0.1098, P = 0.0019
SFA-PG P = 0.9999, P = 0.0914, P = 0.0157
SFA-PI P = 0.0010, P < 0.0001, P < 0.0001
SFA-TAG P = 0.8390, P = 0.4080, P = 0.7288
SFA-FFA P = 0.9700, P = 0.1690, P = 0.7983
ncl-1 RNAi: SFA-PC P = 0.0005, P < 0.0001, P = 0.0007
SFA-PE P = 0.1392, P = 0.0022, P = 0.1371
SFA-PS P = 0.5459, P = 0.0240, P = 0.1523
SFA-PG P = 0.5334, P = 0.0098, P = 0.7720
SFA-PI P = 0.0212, P < 0.0001, P < 0.0001
SFA-TAG P = 0.7409, P = 0.0922, P = 0.5924
SFA-FFA P = 0.0456, P = 0.1795, P = 0.3418

Supplementary Fig. 9 | Differential Pol I activity induces fatty acid composition changes across lipid classes. a, **b**, Ratio of monounsaturated fatty acids (MUFA) to polyunsaturated fatty acids (PUFA) in lipid classes shown as radar plots (**a**) and in PEs shown as bar chart, mean ± S.D. (**b**) in animals treated with Ctrl, *tif-1A* or *ncl-1* RNAi on AD2, AD6 and AD12. **c,** Radar plots showing scores of lipids containing saturated fatty acids (SFA) in animals treated with Ctrl, *tif-1A* or *ncl-1* RNAi on AD2, AD6 and AD12. For **a** and **c** the ratios were calculated from relative intensities and presented as % relative to the control of the respective age. Statistics was computed by two-way ANOVA with Dunnett's post hoc test. For **b**, *P* values comparing *ncl-1* and *tif-1A* RNAi effects to the age-matched Ctrl RNAi baseline were determined using a Student's unpaired *t*-test. ns= not significant, ****$P < 0.0001$, ***$P < 0.001$, ** $P < 0.01$, * $P < 0.05$. In **a**-**c** each condition has five biological replicates of $n = 700$ worms. The raw data and calculations for (**a**) and (**c**) are shown in Supplementary Data 18, for (**b**) – in Supplementary Data 19. All statistical tests used were two-sided, the exact *P* values and statistical analyses are reported in the Source Data file.

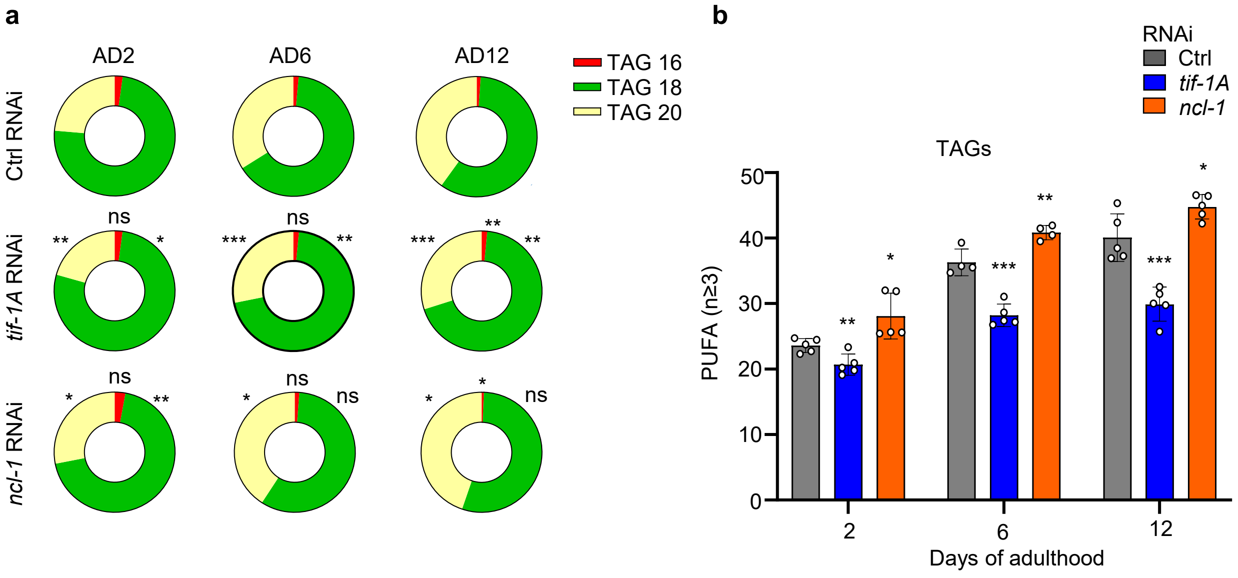

Supplementary Fig. 10 | Differential Pol I activity affects TAG markers of metabolic aging. a, TAG composition in Ctrl RNAi, *tif-1A* RNAi or *ncl-1* RNAi treated animals on AD2, AD6 and AD12 according to the longest chain length among respective fatty acids (TAG 16 to TAG 20), values are expressed as % of total. **b**, Percentage of TAGs with fatty acids containing 3 or more double bonds (n≥3) on AD2, AD6 and AD12 in ctrl RNAi, *tif-1A* RNAi or *ncl-1* RNAi treated animals. Mean ± S.D. values are shown. Raw values (relative intensities) and calculations for (**a**) and (**b**) are presented in Supplementary Data 19. Each condition has five biological replicates of *n* = 700 worms. *P* values comparing *ncl-1* and *tif-1A* RNAi effects to the age-matched Ctrl RNAi baseline were determined using a Student's unpaired *t*-test. ns= not significant, *****P* < 0.0001, ****P* < 0.001, ** *P* <0.01, * *P* <0.05. All statistical tests used were two-sided, the exact *P* values and statistical analyses are reported in the Source Data file.

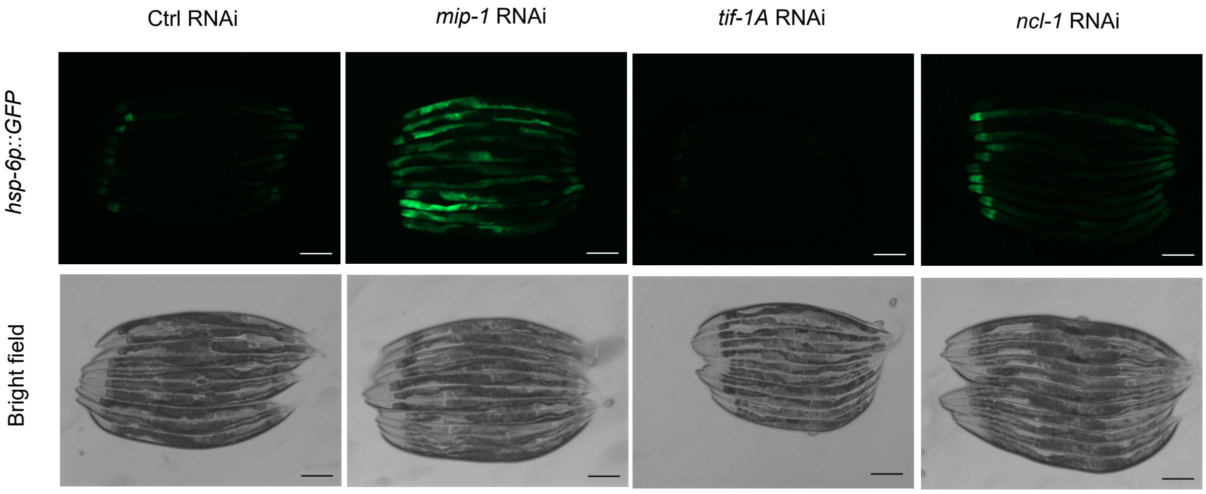

Supplementary Fig. 11 | rDNA activity modulates mitochondrial stress. Transgenic animals expressing GFP under control of the *hsp-6* gene promoter (*hsp-6p*::*GFP (zcls13)*) exposed to Ctrl, *mip-1, ncl-1* or *tif-1A* RNAi were imaged. Scale bar is 200 µm.

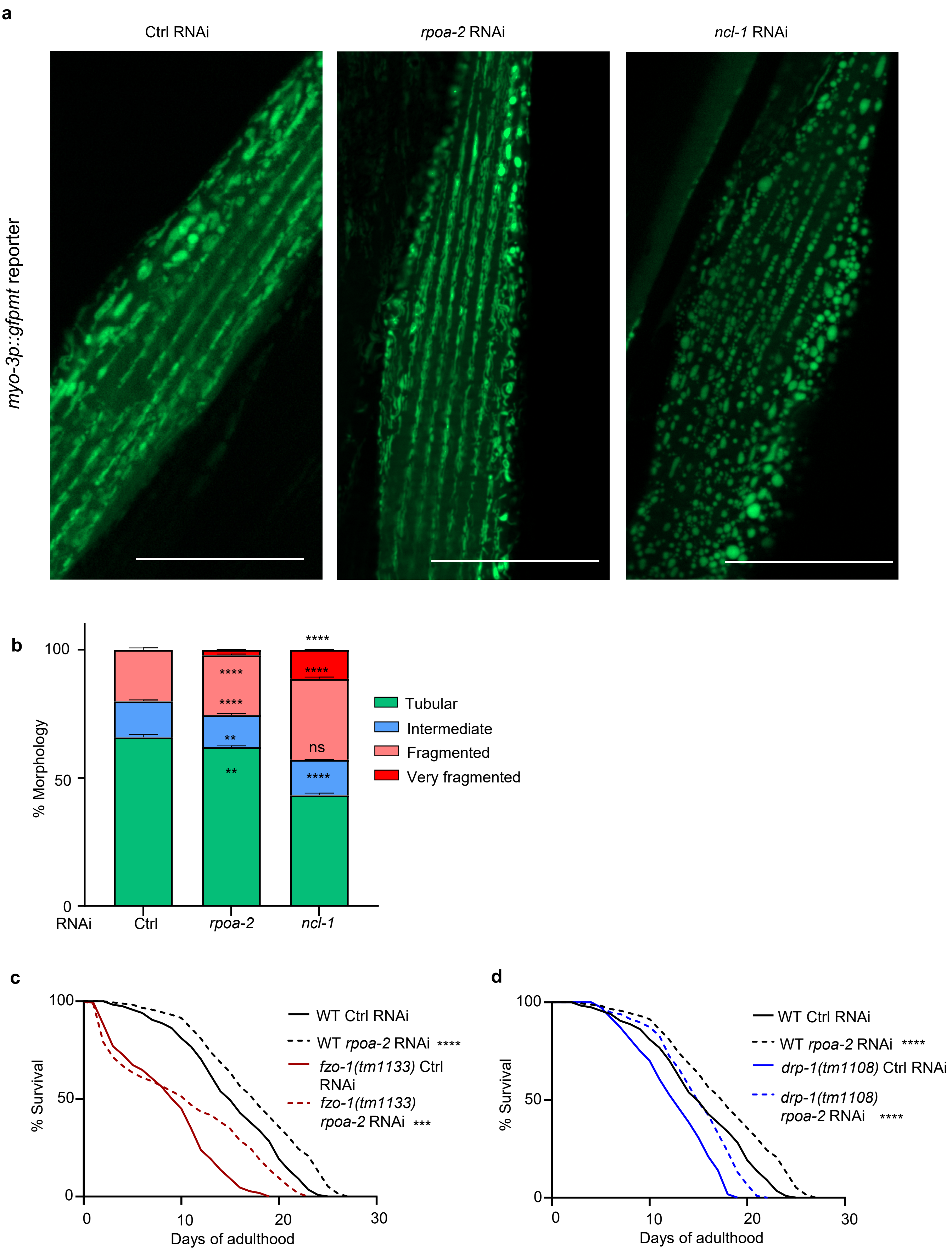

a
Ctrl RNAi
rpoa-2 RNAi
ncl-1 RNAi
myo-3p::gfpmt reporter
b
% Morphology
100
50
0
RNAi
Ctrl
rpoa-2
ncl-1
****
**
ns
Tubular
Intermediate
Fragmented
Very fragmented
c
% Survival
Days of adulthood
WT Ctrl RNAi
WT rpoa-2 RNAi ****
fzo-1(tm1133) Ctrl RNAi
fzo-1(tm1133) rpoa-2 RNAi ***
d
% Survival
Days of adulthood
WT Ctrl RNAi
WT rpoa-2 RNAi ****
drp-1(tm1108) Ctrl RNAi
drp-1(tm1108) rpoa-2 RNAi ****
0
10
20
30

Supplementary Fig. 12 | Life extension by the reduction of Pol I activity is independent of mitochondrial fission or fusion. a, Mitochondrial morphology upon treatment with Ctrl, *rpoa-2* or *ncl-1* RNAi in transgenic nematodes expressing GFP labelled mitochondria in their body wall muscle (*myo-3p::gfpmt (zcls14)*). Representative images of 3 independent trials. Scale bar is 20 μm. **b**, Quantification of mitochondrial morphology. Four distinct morphologies were measured: tubular, intermediate, fragmented and very fragmented. *n*=60 worms per condition. The microscopic analysis was conducted on day 2 of adulthood. Data is mean± SEM. *P* values were determined using Student's unpaired *t*-test with Welch's correction. Results are representative of three independent trials with *n* = 60 worms. **c**, **d**, Survival analyses of wild-type (WT) and *fzo-1(tm1133)* (**b**) or *drp-1(tm1108)* (**c**) nematodes exposed to Ctrl or *rpoa-2* RNAi, *n* = 140. Results are representative of 3 independent trials. *P* values were calculated using Mantel-Cox test. *****P* < 0.0001, ****P* < 0.001, ***P* < 0.01 and **P* < 0.05, ns= not significant. All statistical tests used were two-sided, the exact *P* values and statistical analyses are reported in the Source Data file.

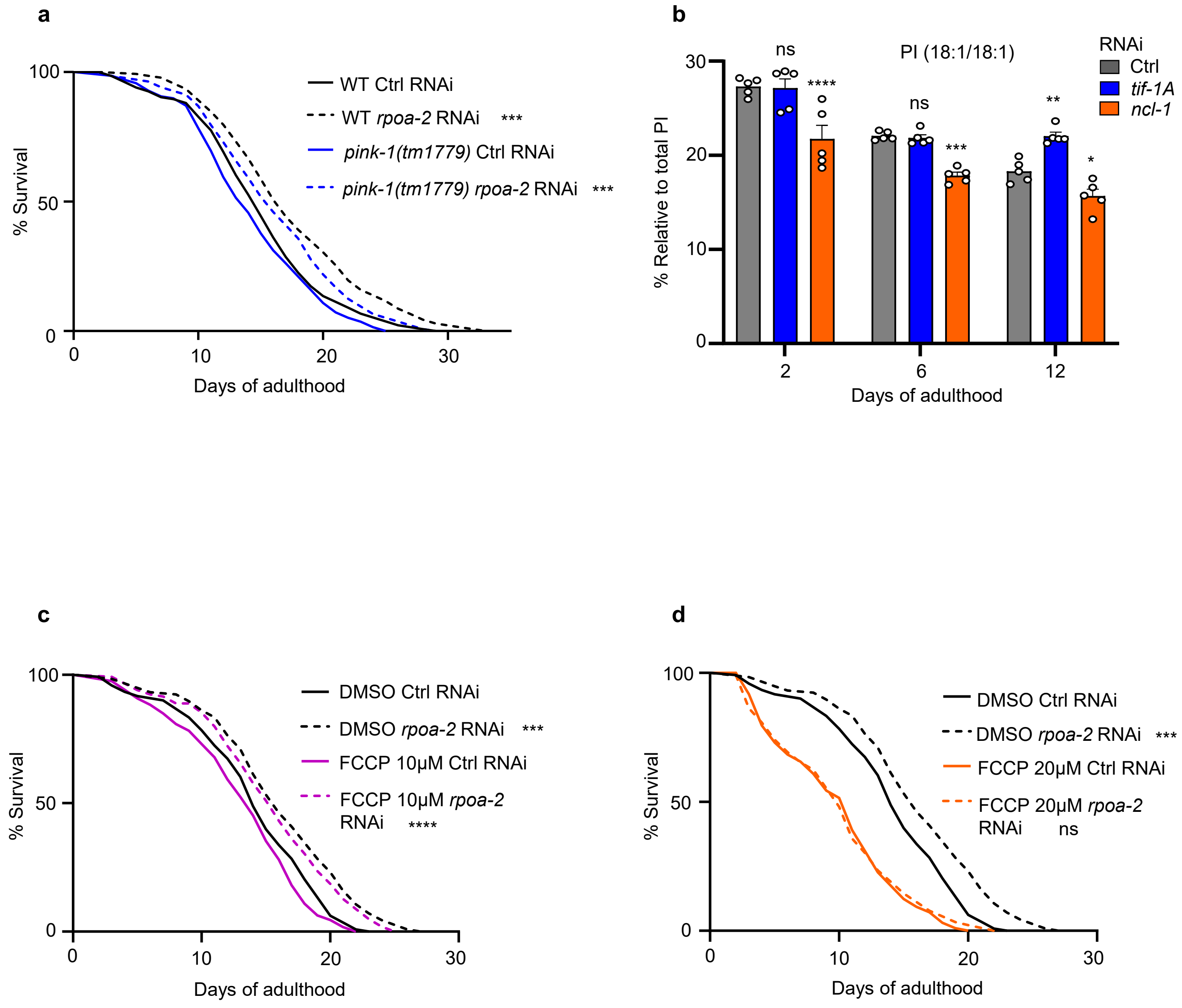

Supplementary Fig. 13 | Reduced Pol I activity exerts lifespan benefits by moderation of mitochondrial stress. a, Survival analyses of wild-type and *pink-1(tm1779)* nematodes exposed to Ctrl or *rpoa-2* RNAi. **b**, Bar chart shows the enrichment of PI(18:1/18:1) lipid on days 2, 6 and 12 of adulthood. Means ± SD values are presented as % of total PI content. Statistical analysis was conducted by two-way ANOVA with Dunnett's post hoc test, each condition has five biological replicates of n = 700 worms. Raw data (relative intensities) and calculations can be seen in Supplementary Data 20. **c, d** Survival analyses of wild-type nematodes treated with DMSO solvent or FCCP 10 µM (**c**) and 20 µM (**d**), and exposed to Ctrl or *rpoa-2* RNAi at 20 °C. In **a**, **c** and **d** survival was scored daily and results representative of 3 independent trials are shown, n = 140 in **a**, and n = 120 in **c**, **d**. P values were calculated using Mantel-Cox test comparing *rpoa-2* RNAi plus and minus conditions in each case. ****$P < 0.0001$, ***$P < 0.001$, ns= not significant. All statistical tests used were two-sided, the exact P values and statistical analyses are reported in the Source Data file.

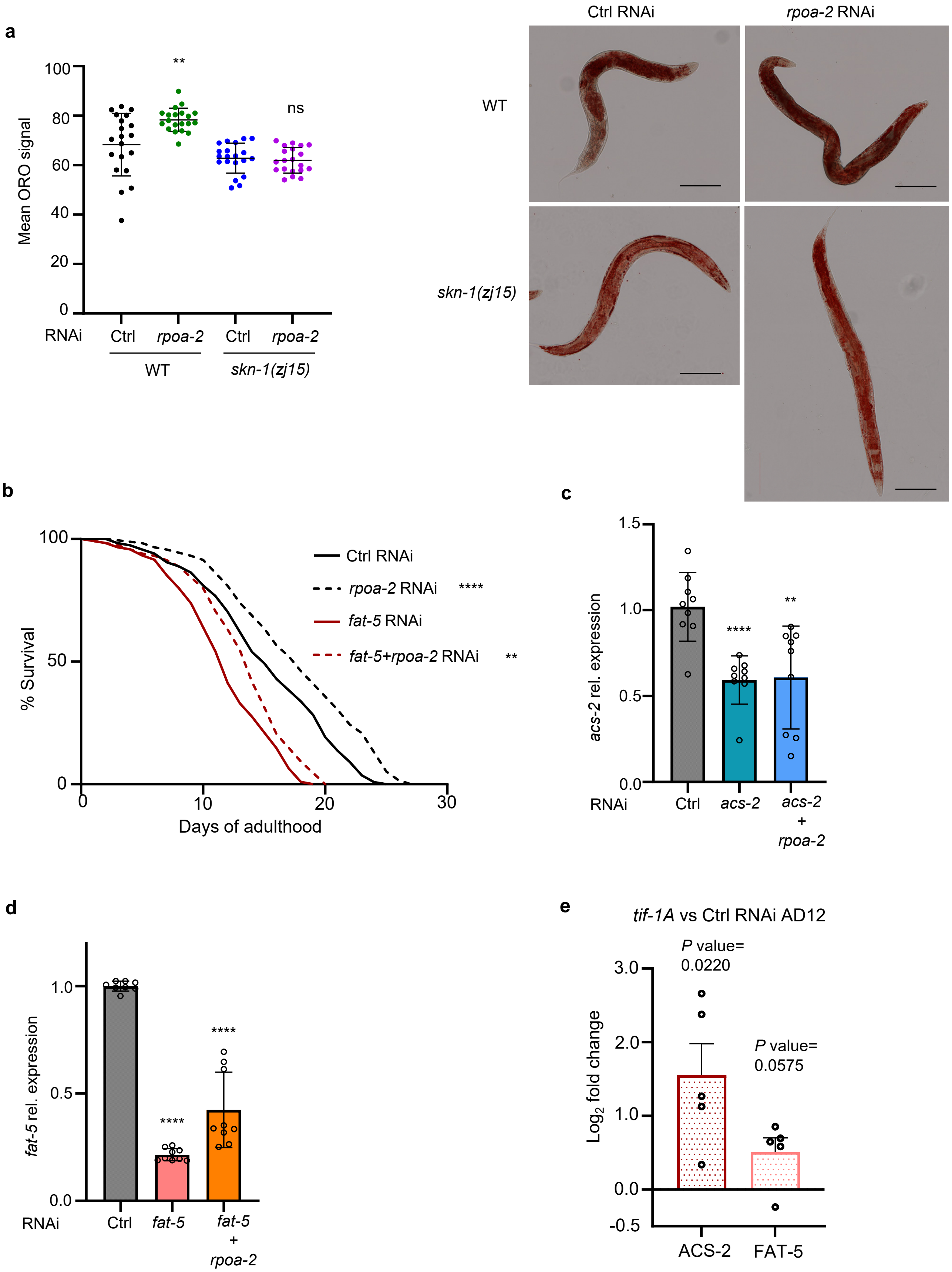

a
Ctrl RNAi
rpoa-2 RNAi
WT
skn-1(zj15)
Mean ORO signal
100
80
60
40
20
0
**
ns
RNAi
Ctrl
rpoa-2
Ctrl
rpoa-2
WT
skn-1(zj15)
b
100
50
0
% Survival
0
10
20
30
Days of adulthood
Ctrl RNAi
rpoa-2 RNAi ****
fat-5 RNAi
fat-5+rpoa-2 RNAi **
c
1.5
1.0
0.5
0.0
acs-2 rel. expression
****
**
RNAi
Ctrl
acs-2
acs-2 + rpoa-2
d
1.0
0.5
0.0
fat-5 rel. expression
****
****
RNAi
Ctrl
fat-5
fat-5 + rpoa-2
e
tif-1A vs Ctrl RNAi AD12
P value= 0.0220
P value= 0.0575
Log2 fold change
3.0
2.0
1.0
0.0
-0.5
ACS-2
FAT-5

Supplementary Fig. 14 | Not all lipidome changes elicited by reduced Pol I activity are required for its lifespan benefits. a, Quantification and representative images of Oil-Red O (ORO) stained wild-type and *skn-1(zj15)* mutant nematodes exposed to Ctrl or *rpoa-2* RNAi. RNAi exposure was initiated at L4 stage and staining was performed on AD6. Each dot represents one worm. Scale bar is 200 μm. Values shown are mean ± S.D. Result representative of 3 trials with *n* = 20 worms. **b**, Survival analysis of wild-type nematodes exposed to Ctrl, *fat-5 and rpoa-2* RNAi as well as *fat-5*+*rpoa-2* RNAi combination. Survival was scored daily, *n* = 120 worms, and statistics was calculated by Mantel-Cox test comparing *rpoa-2* RNAi plus and minus conditions in each case. Result representative of 3 independent trials. **c**, **d**, Expression levels of *acs-2* (**c**) and *fat-5* (**d**) were assessed by RT-qPCR in wild-type nematodes exposed to the indicated RNAi combinations. RNAi exposure was started at L1 stage for *fat-5* and *acs-2,* and at L4 stage for *rpoa-2*, and analysis was performed on AD2. Mean ± S.D. of three independent experiments with *n* = 60 worms each is shown. **e**, Fold change in ACS-2 and FAT-5 protein levels in nematodes exposed to *tif-1A* RNAi vs Ctrl RNAi on AD12 analyzed by LC-MS. Values shown are mean ± S.E.M. of five replicates with *n* = 700 worms each. Statistics for **a**, **c**, **d, e** were calculated using Student's unpaired *t*-test. ns= not significant, *****P* < 0.0001, ***P* < 0.01. Raw data and calculations for (**e**) are shown in Supplementary Data 21. All statistical tests used were two-sided, the exact *P* values and statistical analyses are reported in the Source Data file.

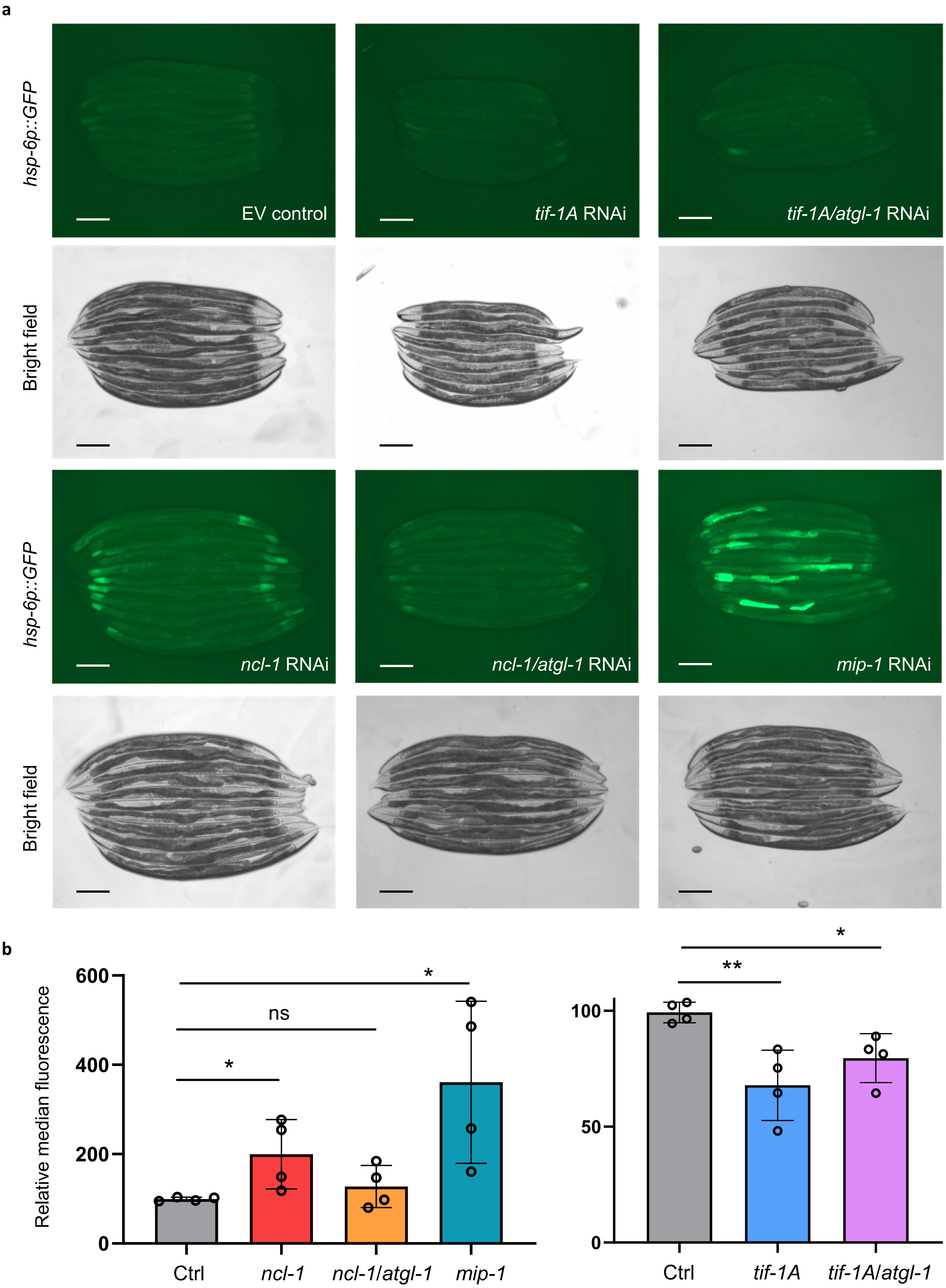
a
hsp-6p::GFP
EV control
tif-1A RNAi
tif-1A/atgl-1 RNAi
Bright field
hsp-6p::GFP
ncl-1 RNAi
ncl-1/atgl-1 RNAi
mip-1 RNAi
Bright field
b
Relative median fluorescence
600
400
200
0
*
ns
*
Ctrl
ncl-1
ncl-1/atgl-1
mip-1
100
50
0
*
**
Ctrl
tif-1A
tif-1A/atgl-1

Supplementary Fig. 15 | Changes of TAG lipolysis link differential Pol I activity to enhanced and reduced mitochondrial stress. **a,** Transgenic animals expressing GFP under control of the *hsp-6* gene promoter (*hsp-6p*::*GFP (zcls13)*) exposed to the indicated RNAi combinations. Scale bar is 200 µm. **b**, Quantification of fluorescence was performed as described in Fig. 5d. Median relative fluorescence intensities of 4 independent experiments recording *n*=10-40 worms per RNAi condition were combined. Values shown are mean± S.E.M. Statistics were calculated using Student's unpaired *t*-test and *P* values are two-tailed. **P* < 0,05; ***P* < 0.01, ns= not significant. The exact *P* values and statistical tests are reported in the Source Data file.

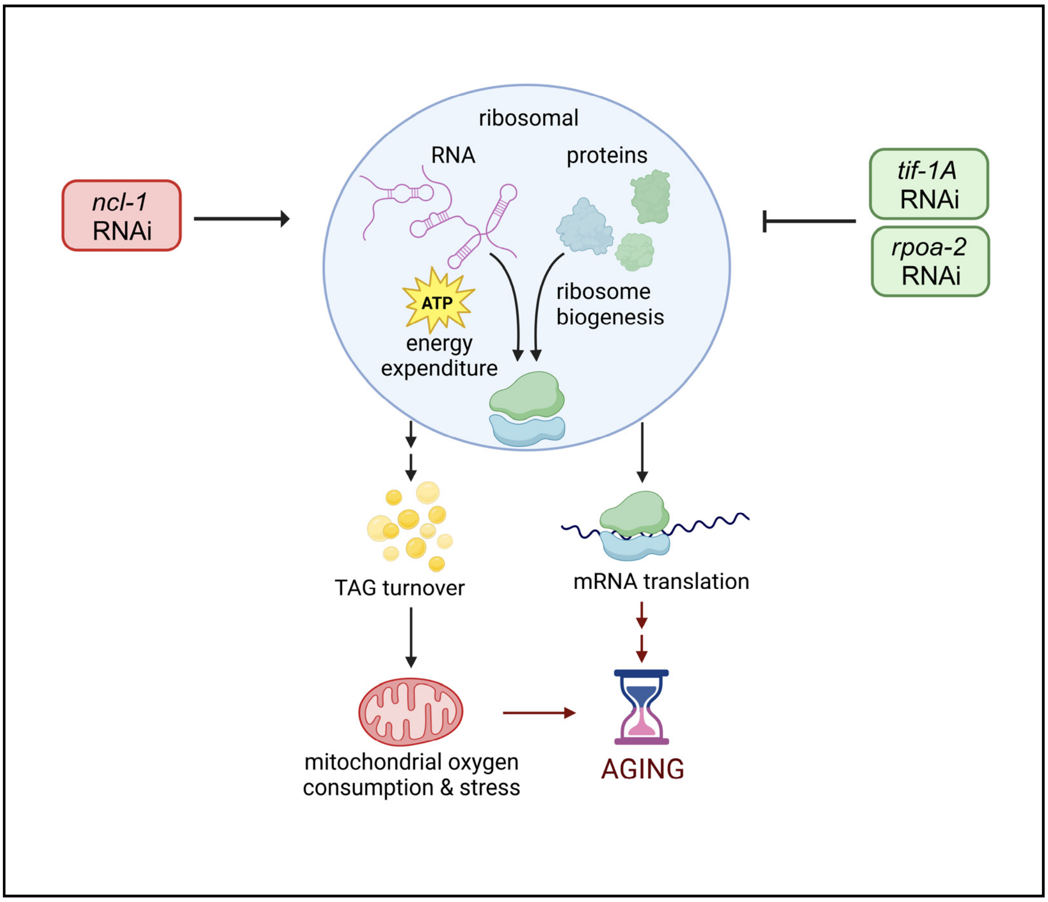

Supplementary Fig. 16 | Model of the mechanistic link between ribosome biogenesis and healthy longevity. The synthesis of rRNA by Pol I is a major driver of highly energy consuming ribosome biogenesis, which in turn fosters mRNA translation. These events apply pressure on the cellular proteostasis machinery, and are known to negatively impact longevity (right branch of the model). Unexpectedly, our work has uncovered a novel metabolic cross-talk between ribosome biogenesis, lipid metabolism and mitochondrial stress. We show that Pol I activity and ribosome biogenesis are key modulators of cellular ATP expenditure. Subsequently, we find that elevated ribosome production triggers catabolism of energy-storage triacylglycerides (TAGs), which forces mitochondrial oxygen consumption. The resulting stress and damage of mitochondria is detrimental to longevity (left branch of the model). Accordingly, genetic interventions that diminish (*tif-1A* and *rpoa-2* RNAi) or enhance (*ncl-1* RNAi) rRNA synthesis protract or accelerate aging, respectively. Image was created with BioRender.com.

a

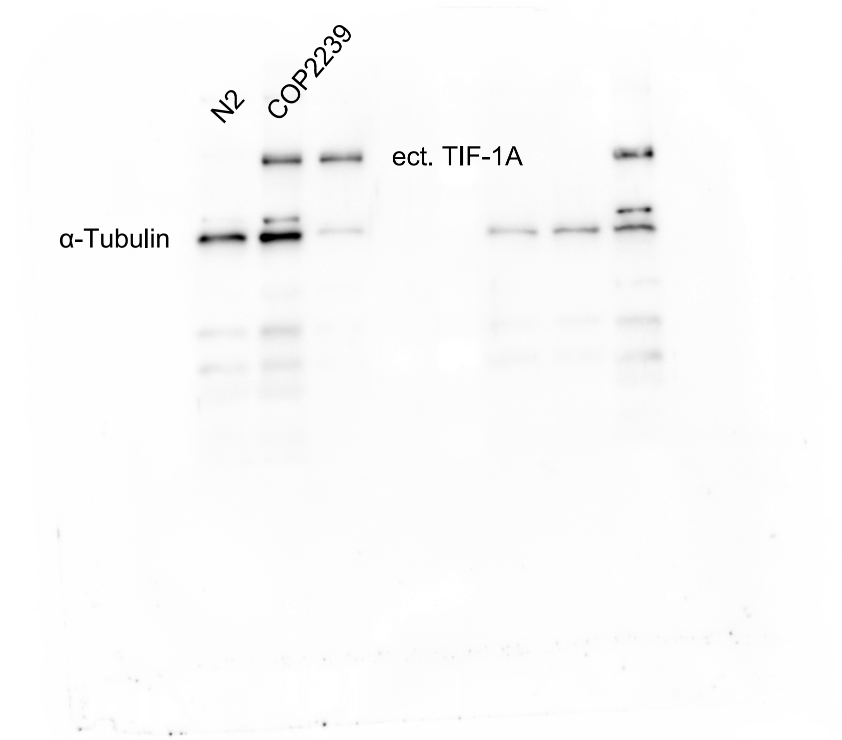

b

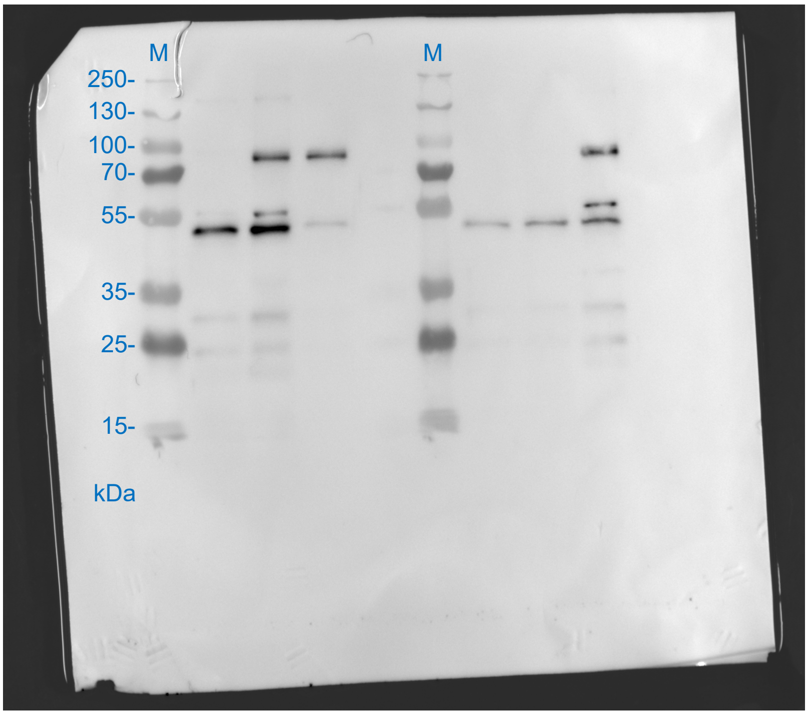

Supplementary Fig. 17 | Original blot scans for Supplementary Figure 1a. The antibody staining is shown in (**a**) and merge with the molecular weight marker and membrane is shown in (**b**). The blot contains multiple analyses, but only strains relevant for this study are labelled.
