# Supplementary material for: Reducing the metabolic burden of rRNA synthesis promotes healthy longevity in Caenorhabditis elegans
Source: Nat Commun. 2024 Feb 24;15:1702. doi: 10.1038/s41467-024-46037-w (PMC10894287; doi:10.1038/s41467-024-46037-w)
Supplement: Supplementary file 3 — Description of Additional Supplementary Files [file 41467_2024_46037_MOESM3_ESM.pdf]

Description of Additional Supplementary Files

Title: Supplementary Data 1

Description: Proteomics raw comparisons, complete dataset.

Title: Supplementary Data 2

Description: Selected proteomics comparisons used in Figure 3a.

Title: Supplementary Data 3

Description: Selected proteomics comparisons used in Figure 3b.

Title: Supplementary Data 4

Description: Input for the WormCat analysis of proteins significantly regulated between old *tif-1A* knockdown and control cohorts.

Title: Supplementary Data 5

Description: WormCat output corresponding to Supplementary Figure 4.

Title: Supplementary Data 6

Description: Input for the WormCat analysis of proteins significantly regulated between old *ncl-1* knockdown and control cohorts.

Title: Supplementary Data 7

Description: WormCat output corresponding to Supplementary Figure 5.

Title: Supplementary Data 8

Description: WormCat output corresponding to Figure 3c.

Title: Supplementary Data 9

Description: WormCat output corresponding to Figure 3d.

Title: Supplementary Data 10

Description: Selected proteomics comparisons corresponding to Figure 3e.

Title: Supplementary Data 11

Description: Selected proteomics comparisons corresponding to Figure 3f.

Title: Supplementary Data 12

Description: Lipidomics raw data (absolute intensities), and analysis corresponding to Figure 5a.

Title: Supplementary Data 13

Description: Lipidomics raw data (relative intensities) corresponding to Figure 5b.

Title: Supplementary Data 14

Description: Log_{10}-transformed lipidomics data corresponding to Figure 5b, left panel, derived from the raw data shown in Supplementary Data 13. Relevant calculations are presented.

Title: Supplementary Data 15

Description: Log_{10}-transformed lipidomics data corresponding to Figure 5b, right panel, derived from the raw data shown in Supplementary Data 13. Relevant calculations are presented.

Title: Supplementary Data 16

Description: Lipidomics raw data (relative intensities) and calculations corresponding to Supplementary Figure 8a.

Title: Supplementary Data 17

Description: Lipidomics raw data (absolute intensities) and calculations corresponding to Supplementary Figures 8b-g.

Title: Supplementary Data 18

Description: Lipidomics raw data (relative intensities) and calculations corresponding to Supplementary Figures 9a and 9c.

Title: Supplementary Data 19

Description: Complete relative intensities lipidomics raw data and calculations corresponding to Figure 5c, and Supplementary Figures 9b, 10a and 10b.

Title: Supplementary Data 20

Description: Lipidomics raw data (relative intensities) and calculations corresponding to Supplementary Figure 13b.

Title: Supplementary Data 21

Description: Selected proteomics comparisons corresponding to Supplementary Figure 14e.

Title: Supplementary Data 22

Description: All PCR primer sequences used in this study.

Title: Supplementary Data 23

Description: Summary of incubation temperatures and RNAi exposure durations for all *C. elegans* experiments performed in this study.
