# Supplementary material for: Reducing the metabolic burden of rRNA synthesis promotes healthy longevity in Caenorhabditis elegans
Source: Nat Commun. 2024 Feb 24;15:1702. doi: 10.1038/s41467-024-46037-w (PMC10894287; doi:10.1038/s41467-024-46037-w)
Supplement: Supplementary file 5 — Reporting Summary [file 41467_2024_46037_MOESM5_ESM.pdf]

nature portfolio

Corresponding author(s): Maria Ermolaeva, Holger Bierhoff

Last updated by author(s): Jan 23, 2024

Reporting Summary

Nature Portfolio wishes to improve the reproducibility of the work that we publish. This form provides structure for consistency and transparency in reporting. For further information on Nature Portfolio policies, see our Editorial Policies and the Editorial Policy Checklist.

Statistics

For all statistical analyses, confirm that the following items are present in the figure legend, table legend, main text, or Methods section.

n/a	Confirmed	
☐	☒	The exact sample size (*n*) for each experimental group/condition, given as a discrete number and unit of measurement
☐	☒	A statement on whether measurements were taken from distinct samples or whether the same sample was measured repeatedly
☐	☒	The statistical test(s) used AND whether they are one- or two-sided<br>*Only common tests should be described solely by name; describe more complex techniques in the Methods section.*
☐	☒	A description of all covariates tested
☐	☒	A description of any assumptions or corrections, such as tests of normality and adjustment for multiple comparisons
☐	☒	A full description of the statistical parameters including central tendency (e.g. means) or other basic estimates (e.g. regression coefficient) AND variation (e.g. standard deviation) or associated estimates of uncertainty (e.g. confidence intervals)
☐	☒	For null hypothesis testing, the test statistic (e.g. *F*, *t*, *r*) with confidence intervals, effect sizes, degrees of freedom and *P* value noted<br>*Give P values as exact values whenever suitable.*
☒	☐	For Bayesian analysis, information on the choice of priors and Markov chain Monte Carlo settings
☒	☐	For hierarchical and complex designs, identification of the appropriate level for tests and full reporting of outcomes
☒	☐	Estimates of effect sizes (e.g. Cohen's *d*, Pearson's *r*), indicating how they were calculated

Our web collection on statistics for biologists contains articles on many of the points above.

Software and code

Policy information about availability of computer code

Data collection	Data collection was performed as previously described (Espada et al, Nature Metabolism, 2020)
Data analysis	Data analysis was performed as previously described (Espada et al, Nature Metabolism, 2020) in most of the cases. In addition, WormCat 2.0 gene set enrichment analysis tool was used to analyze proteomics data. Excel and GraphPad Prism 8.4.3 were used for numerical analyses and figure preparation. ZEN 3.1 (blue addition) software (Carl Zeiss) was used for image analyses. DIA raw data (proteomics) were analyzed using the directDIA pipeline in Spectronaut v.13 (Biognosysis AG). Lipidomics data was assessed using Analyst 1.6.2 (Sciex).

For manuscripts utilizing custom algorithms or software that are central to the research but not yet described in published literature, software must be made available to editors and reviewers. We strongly encourage code deposition in a community repository (e.g. GitHub). See the Nature Portfolio guidelines for submitting code & software for further information.

Data

Policy information about availability of data

All manuscripts must include a data availability statement. This statement should provide the following information, where applicable:

- Accession codes, unique identifiers, or web links for publicly available datasets
- A description of any restrictions on data availability
- For clinical datasets or third party data, please ensure that the statement adheres to our policy

Proteomics data were searched against a species-specific (C.elegans, 26.677 entries) Swissprot database.The mass spectrometry proteomics data, including the

exact UniProt information, have been deposited to the ProteomeXchange Consortium via the PRIDE77 partner repository with the dataset identifier PXD028600. The data can be accessed at http://www.ebi.ac.uk/pride/archive/projects/PXD028600. The mass spectrometry lipidomics data generated in this study have been deposited in the Metabolomics Workbench database (an international repository for metabolomics data and metadata, metabolite standards, protocols, tutorials and training, and analysis tools) under Project ID PR001490 (http://dx.doi.org/10.21228/M84D89).

Research involving human participants, their data, or biological material

Policy information about studies with human participants or human data. See also policy information about sex, gender (identity/presentation), and sexual orientation and race, ethnicity and racism.

Reporting on sex and gender	NA
Reporting on race, ethnicity, or other socially relevant groupings	NA
Population characteristics	NA
Recruitment	NA
Ethics oversight	NA

Note that full information on the approval of the study protocol must also be provided in the manuscript.

Field-specific reporting

Please select the one below that is the best fit for your research. If you are not sure, read the appropriate sections before making your selection.

☒ Life sciences ☐ Behavioural & social sciences ☐ Ecological, evolutionary & environmental sciences

For a reference copy of the document with all sections, see nature.com/documents/nr-reporting-summary-flat.pdf

Life sciences study design

All studies must disclose on these points even when the disclosure is negative.

Sample size	Sample size for each assay was determined individually. In most cases, the choice was based on previous relevant publications. For the survival analysis of C. elegans 2 cohorts of 70 animals were used per condition to achieve n over 100 at the end of the test, accounting for the spontaneous loss of animals during the experiment (Petrascheck and Miller, Front Genet 2017). For ATP measurements 2 cohorts of 50 animals (n=100 in total) were used based on the previous report (Palikaras et al, Nature 2015). For proteomics and lipidomics analysis 700-800 animals were chosen as optimal sample size based on previous successful tests (Espada et al, Nat Metabolism, 2020). For microscopy experiments (mitochondrial UPR induction, Oil Red O staining and mitochondrial morphology tests) the animal numbers were chosen on the basis of previous reports (Espada et al, Nat Metabolism, 2020 and Burkewitz et al, Cell 2016 respectively). Sample size for in vivo respirometry tests was defined as in Espada et al 2020. For western blot analysis the same rationale was adopted as in proteomics tests. For gene expression assays the optimal sample size was established experimentally based on sufficient quantity and quality of extracted RNA. For locomotory and smurf assays the numbers were guided by previous publications describing these methods (Lesanpezeshki L et al, Sci Rep, 2019 and Gelino S et al, Plos Genet, 2016). The exact sample size and number of independent replicas for all presented tests is reported in the legends and the Statistics Source Data file.
Data exclusions	Censoring of C. elegans was applied according to standard procedures (Zhao et al, Nat Commun 2017), specifically contaminated plates, spontaneously ruptured animals, bag of worms phenotype, missing animals and animals damaged during handling were noted as censored.
Replication	Each figure is based on at least 3 independent experiments or replicates; only experiments that showed reproducible results were considered. Figures show either representative results or a summary of all trials, as specified in the respective legends.
Randomization	To achieve randomization, respective C. elegans strains were subjected to bleaching, yielding thousands of age-synchronized L1 larvae. For proteomics and lipidomics tests, 700 worms were randomly transferred from these larger cohorts onto replica plates, 5 replicas per condition, and maintained as independent cultures until sample collection. For survival experiments, 500-600 animals were randomly allocated from the larger L1 cohorts and subjected to designated RNAi and other treatments. At the L4 stage, 2 random cohorts of 70 animals were transferred from these pre-cultures onto 2 separate 60 mm dishes for survival observation. The use of 2 independent plates was needed to account for potential plate-to-plate variations. For ATP testing, 500 animals were randomly taken from the larger L1 cohorts and subjected to designated RNAi and other treatments. On the day of the analysis, 1-4 cohorts of 50 animals were taken onto separate 60 mm dishes without bias and analyzed independently. For microscopy assays, a similar randomization procedure was followed with intermediate post-L1 and pre-analysis cultures varying in size. For mitochondrial stress assay, the intermediate culture contained 300-500 animals, 2 cohorts of 50 animals were randomly allocated for imaging and 10-25 were imaged. For mitochondrial morphology assay, the size of intermediate culture was 300-400 animals, 3 cohorts of 30 animals were randomly picked onto slides and 20 animals per slide were imaged. For locomotory assay, 3 random cohorts of 30 animals were scored from an intermediate culture of 500 animals. For smurf assay, 40-60 worms per condition were randomly taken from intermediate cultures of 500 animals, stained and scored. For nucleolar morphology measurement 20 animals per condition were placed on 2 independent slides (10 animals in one slide) from intermediate cultures of 500 worms; each animal was assessed individually. For cell culture tests, frozen cell cultures were thawed and seeded on plates without bias for further treatment and analysis.

Blinding | In this study we used assays that do not entail significant risks of the researcher bias. For instance, proteomics and lipidomics data were automatically acquired and processed by high precision instruments and software. The intensities of ORO and GFP signals were assessed by the dedicated image analysis software with minimal and highly standardized input by the researcher. Respirometry assays were automated and ATP and MTT measurements relied on values recorded by the automated plate reader device. Gene expression values were delivered by a qPCR device. In summary, many of the assays relied on fully- or semi-automated data acquisition thereby minimizing researcher bias. At the same time, non-automated assays such as locomotory, smurf or survival assays were based on clear binary outcomes (living/dead, dye staining only intestine or whole animal, quantification of all animals visible on the plate surface at a given time) again minimizing the possibility of unconscious bias. Because of these considerations, blinding was not considered necessary and was not applied.

Reporting for specific materials, systems and methods

We require information from authors about some types of materials, experimental systems and methods used in many studies. Here, indicate whether each material, system or method listed is relevant to your study. If you are not sure if a list item applies to your research, read the appropriate section before selecting a response.

Materials & experimental systems

n/a	Involved in the study
☐	☒ Antibodies
☐	☒ Eukaryotic cell lines
☒	☐ Palaeontology and archaeology
☐	☒ Animals and other organisms
☒	☐ Clinical data
☒	☐ Dual use research of concern
☒	☐ Plants

Methods

n/a	Involved in the study
☒	☐ ChIP-seq
☒	☐ Flow cytometry
☒	☐ MRI-based neuroimaging

Antibodies

Antibodies used | Primary antibodies used were: anti-Ty1 (Diagenode, Cat# C15200054, Lot# 007) and anti-α-Tubulin (Clone DM1A, Merck KGaA, Cat# T6199, Lot# 0000127611). The working dilution was 1:5000 for both primary antibodies. Secondary anti-mouse antibody (Dianova, Cat# DkxMu-003-FHRPX, Lot# 67-83-071919) was used with working dilution of 1:10000.

Validation | The antibodies were used for Western blotting in Figure S1a according to manufacturer's instructions. As stated by manufacturers, both antibodies were previously validated for this application.

Eukaryotic cell lines

Policy information about cell lines and Sex and Gender in Research

Cell line source(s) | BJ human foreskin fibroblasts were purchased from ATCC (Reference number CRL-2522)

Authentication | BJ cell line was authenticated and tested by ATCC using morphology, karyotyping and PCR based approaches. Link: https://www.lgcstandards-atcc.org/CellAuthenticationMatters

Mycoplasma contamination | BJ cell line was proved free from mycoplasma contamination by Mycoplasma PCR ELISA (Sigma Aldrich)

Commonly misidentified lines (See ICLAC register) | No misidentified cell lines were used in this study

Animals and other research organisms

Policy information about studies involving animals; ARRIVE guidelines recommended for reporting animal research, and Sex and Gender in Research

Laboratory animals | Age-synchronized wild type and mutant C. elegans hermaphrodites were used in our tests. The C. elegans wild isolate (Bristol N2), ife-2(ok306) mutant (KX15), atfs-1(gk3094) mutant (VC3201), pink-1(tm1779) mutant (BR4006, without transgen), skn-1(zj15) mutant (QV225), prx-5(ku517) mutant (MH5239), myo-3p::gfpmt (zcls14) expressing strain (SJ4103), hsp-6p::GFP expressing strain (SJ4100) and FIB 1::GFP expressing strain (COP262) were obtained from the NIH funded Caenorhabditis Genetics Centre (CGC). The tif-1A overexpressing strain COP2239 (genotype: 3p::3xTy1::C36E8.1::tbb-2u in ttTi5605, unc-119(+)) II ; unc 119(ed3) III) was generated by InVivo Biosystems using the MosSCI method. Animals were handled by using established methods and guidelines for C. elegans aging research. Young (adulthood day 2), middle aged (adulthood day 6) and old (adulthood day 12) animals were tested.

Wild animals | Wild animals were not used in this study

Reporting on sex | Hermaphrodite animals were used in all C. elegans tests

Field-collected samples	Field-collected samples were not used in this study
Ethics oversight	This study didn't involve materials or animal models that require ethical approval

Note that full information on the approval of the study protocol must also be provided in the manuscript.

Plants

Seed stocks	NA
Novel plant genotypes	NA
Authentication	NA
